# The case for a global therapeutics development coalition: Building a therapeutics pipeline for pandemic and endemic diseases

**DOI:** 10.1371/journal.pgph.0003654

**Published:** 2024-08-30

**Authors:** Shingai Machingaidze, Carmen Pérez Casas, Sheila Mburu, Ruxandra Draghia-Akli, Charles Mowbray, James Rosen, Esteban Burrone, Mona Nemer, Victor Dzau

**Affiliations:** 1 International Pandemic Preparedness Secretariat (IPPS), London, United Kingdom; 2 Unitaid, Geneva, Switzerland; 3 International Readiness for Preventing Infectious Viral Disease (INTREPID Alliance), Cambridge, Massachusetts, United States of America; 4 Drugs for Neglected Diseases initiative (DNDi), Geneva, Switzerland; 5 Rapidly Emerging Antiviral Drug Development Initiative (READDI), Chapel Hill, North Carolina, United States of America; 6 Medicines Patent Pool (MPP), Geneva, Switzerland; 7 Office of the Chief Science Advisor, Government of Canada, Ottawa, Canada; 8 National Academy of Medicine, Washington DC, United States of America; PLOS: Public Library of Science, UNITED STATES OF AMERICA

## Why do therapeutics matter?

Safe and effective therapeutics, together with other life-saving tools, are critical to an effective outbreak response. Therapeutics are also used for treating endemic diseases, reducing mortality and morbidity and health system burden. The deployment of effective therapeutics for infectious diseases is particularly essential while vaccines are being developed, tested and rolled out, or for diseases for which vaccine development is challenging and lengthy. The COVID-19 pandemic starkly highlighted the underdeveloped and poorly coordinated pipeline of safe, effective, fit-for-purpose, and affordable therapeutics for diseases with pandemic potential, with the first oral antivirals becoming available 20 months after the first COVID-19 vaccines [1], which were authorised for use in under a year [2]. In future health emergencies we may not be so lucky, meaning therapeutics may well be all we have. So how do we ensure a healthy global therapeutics pipeline and equitable access to low-and-middle income countries?

Lessons learnt from previous outbreaks, and the panic-neglect cycle, demonstrate the importance of a sustainable and responsive end-to-end research and development (R&D) ecosystem, which can be mobilised during emergencies to rapidly develop key medical countermeasures (MCMs)–including therapeutics [3]. The 100 Days Mission [4], welcomed by G7 and G20 leaders in 2021, aims to strengthen global pandemic preparedness, and highlights the need to ensure that therapeutics (alongside diagnostics and vaccines) are authorized for use within 100 days of a Public Health Emergency of International Concern (PHEIC) being declared. However, among WHO’s priority pathogens with pandemic potential [5], there are currently only approved therapeutics available for COVID-19 and Ebola Zaire [6] and few candidates for other viral families of concern progressing through the development pipeline [7]. In addition, there is limited availability of antiviral products for endemic diseases. Furthermore, even when therapeutics become available, access is not equitable–particularly in LMICs, as seen in the poor access to nirmatrelvir-ritonavir. Addressing critical gaps in antiviral therapeutics development is a vital next step to drive improvements in global health and to strengthen our ability to respond equitably to future health threats [1].

## Gaps and challenges in the development of therapeutics: Equity must be at the centre

A critical lack of funding for therapeutics R&D, before and during the recent pandemic, hampered efforts to develop a timely therapeutic armamentarium for COVID-19, beyond a few exceptions mostly to treat the most severe cases with little impact in the pandemic evolution [1]. Limited and uncoordinated investments for R&D during the pandemic rendered most of the scarce efforts fragmented, and/or underpowered and duplicative, both in the development and evaluation of the emerging candidates, with the exception of a few rapidly developed clinical trial initiatives, such as the US National Institutes of Health Accelerating COVID-19 Therapeutic Interventions and Vaccines (ACTIV) public-private partnership [8], the Oxford University-led clinical trial RECOVERY [9], the World Health Organisation (WHO) led Solidarity trials [10] and ANTICOV [11].

During the first year of the COVID-19 pandemic, 20 times less was spent globally on therapeutics R&D compared to vaccines ($4.6bn versus $91bn), and the therapeutics pillar of the Access to COVID-19 Tools (ACT) Accelerator received less than 10% of donor funding, compared to nearly 70% allocated to COVID-19 Vaccines Global Access (COVAX) for vaccines [6].

End-to-end development costs for new anti-viral therapeutics can be as high as $800m-$2.5bn [6]. While predicting demand is difficult, collaborative public-private partnership models can help to significantly reduce costs [12]. Market forces alone are unlikely to incentivise investment in antiviral therapeutics R&D. At present, neither industry nor governments are investing sufficiently, leading to a relatively bare therapeutics pipeline. There is an opportunity for diverse stakeholders to unite around a clear vision, and coordinated approach on how to direct their resources towards reinvigorating the therapeutics pipeline–exploring how to incentivise and de-risk investment in end-to-end therapeutics R&D.

Increased, coordinated, and sustained investment to develop therapeutics that can be rapidly deployed, is a pivotal next step, with equitable access as a key principle from development to delivery. Equitable access to MCMs remains a challenge, especially for LMICs [4, 13]. During the 2014–16 West Africa Ebola outbreak for example, experimental monoclonal antibodies were not available in endemic regions [14]. Following this outbreak however, stakeholders in the R&D ecosystem worked together to develop Ebola vaccines and therapeutics, with new monoclonal antibodies subsequently recommended for use by WHO. Despite this, products take time to become available for use in disease-endemic countries experiencing new Ebola outbreaks [3]. Equitable access must be embedded throughout the therapeutics development value chain [1], and we must ensure that products are accessible in countries that need them, and are not solely in high-income country stockpiles.

## Leveraging global stakeholders to build a therapeutics development coalition: A path forward

To invigorate the therapeutics pipeline for future pandemics and endemic disease, and drive advocacy for increased investment and coordination of end-to-end therapeutics development, there is a need to bring together a coalition of stakeholders across academia, early-stage developers, government, international organisations, civil society, and private sector. Rather than creating a new entity, this should be a genuine coalition of existing partners working towards the shared goal of ensuring availability and access to therapeutics. A recent meeting held at the Wellcome Trust, London (June 2024) (S1 Table) marked the beginning of this collective endeavour, bringing together stakeholders from across the world to discuss what is needed to make this a reality across the therapeutics value chain, recognising the primary gap to address is the dearth of candidates in the pipeline.

There was consensus on the need for an independent and inclusive coalition to reinvigorate the therapeutics pipeline, and willingness to collaborate on end-to-end therapeutics development, and platform technologies and tools that can enable such a pipeline to be more efficient, for both endemic and pandemic pathogens, ensuring alignment with WHO’s work on R&D for priority viral families [5], coordination mechanism for equitable access to medical countermeasures (iMCM-Net) [15], and WHO global action plan to strengthen the clinical trial ecosystem.

**Fig 1:**
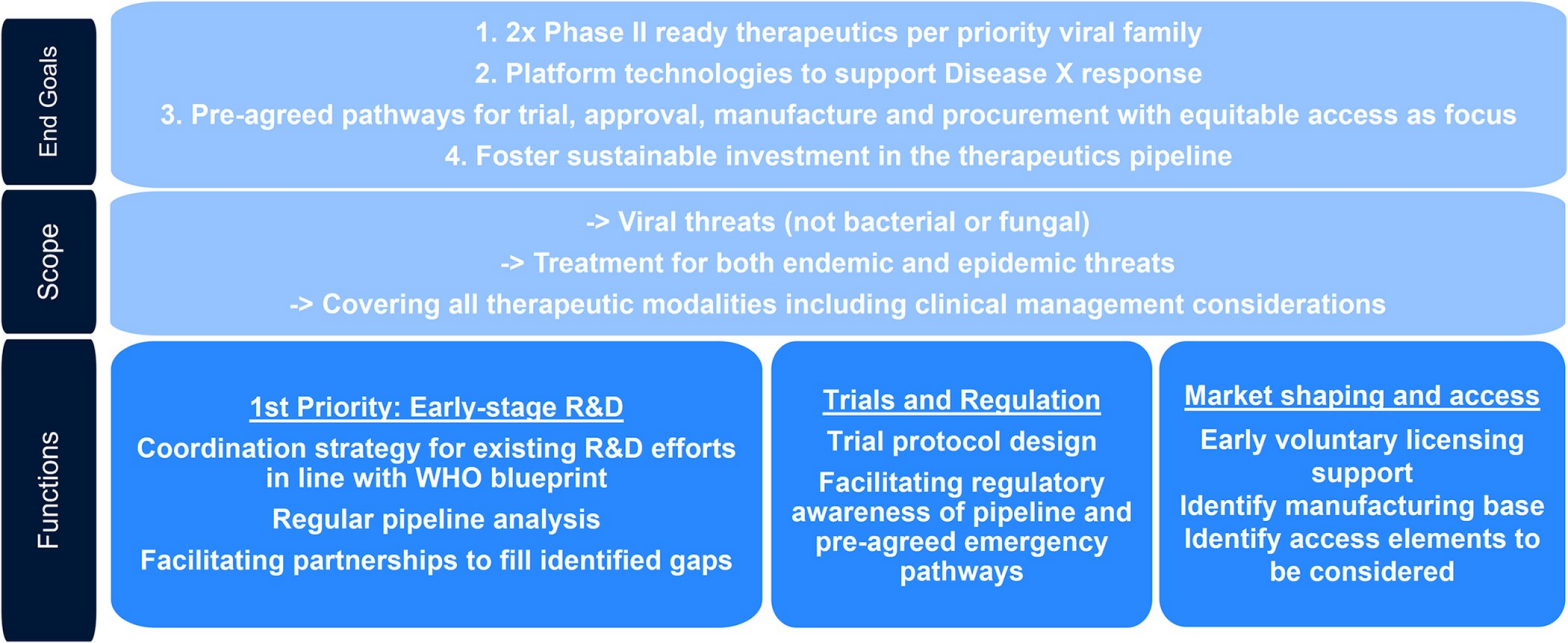
Proposed scope, goals and function of a therapeutics development coalition.

The coalition will support the goals of ensuring two phase-2 ready therapeutics for viral pandemic pathogens [6] and candidates for endemic diseases, with a focus on priority families and broad-spectrum compounds and tools (Fig 1). It will also aim to develop scientifically rigorous and validated programmable platform technologies capable of speeding up the availability of new therapeutics, or enhancing existing therapeutics for endemic and pandemic diseases, and “Disease X”. Importantly, to ensure access is built at early stages of the R&D pipeline, it will aim to establish pre-agreed pathways and templates for clinical trials (including use of already established clinical trials networks), regulation, manufacturing and procurement and advocating for investments. Ensuring sustained R&D funding throughout the development lifecycle will be critical.

The ultimate long-term goal of this global therapeutics’ development coalition is to facilitate rapid, equitable access to pandemic and endemic therapeutics enabled by a strong product pipeline, supported by flexible supply capacity, optimised manufacturing, established regulatory pathways and market shaping activities. We invite interested stakeholders to consider how they can contribute. Developers and those in early-stage research are invited to express interest in joining this collaborative approach to avoid duplication and share resources, public or private funders to consider increasing investment in R&D, and governments and procurers to consider market signals they could send to invigorate the market.

## Supporting information

S1 Table List of organisations at meeting entitled towards building a therapeutics coalition (in-person and online). (DOCX)
