# Supplementary material for: The case for a global therapeutics development coalition: Building a therapeutics pipeline for pandemic and endemic diseases
Source: PLOS Glob Public Health. 2024 Aug 30;4(8):e0003654. doi: 10.1371/journal.pgph.0003654 (PMC11364229; doi:10.1371/journal.pgph.0003654)
Supplement: S1 Table — (DOCX) [file pgph.0003654.s001.docx]

*Table 1: Organisations at meeting – Towards building a Therapeutics Coalition (in person and online)*

| Africa Medical Supplies Platform |
| --- |
| ANRS Emerging Infectious Diseases |
| AI -driven Structure-enabled Antiviral Platform (ASAP) NIH Antiviral Drug Discovery (AViDD) Centre |
| ASAP Discovery Consortium |
| Bio-Manguinhos |
| Biomedical Advanced Research and Development Authority (BARDA) |
| Canadian Pandemic Preparedness Hub |
| Center for Strategic and International Studies (CSIS) |
| Clinton Health Access Initiative |
| Coalition for Epidemic Preparedness (CEPI) |
| COVID Collaborative |
| Cumming Global Centre for Pandemic Therapeutics |
| Drugs for Neglected Disease Initiative (DNDi) |
| Doherty Institute, University of Melbourne |
| Duke University/ Duke Global Innovation Centre |
| Evotec |
| Foundation for Innovative New Diagnostics (FIND) |
| Foundation for National Institutes of Health |
| Foundation for National Institutes of Health (FNIH) |
| Global Health Investment Corporation (GHIC) |
| Gilead Sciences Inc |
| GlaxoSmithKline |
| European and Developing Countries Clinical Trials Partnership – Global Health EDCTP3 |
| Government of Canada |
| Drug Discovery and Development Centre (H3D) Africa |
| Harvard Medical School \| Brigham & Women's Hospital |
| House of Lords |
| Institute de Recherches Cliniques de Montreal (ICRM) |
| International Federation of Pharmaceutical Manufacturers and Associations (IFPMA) |
| Institute for Medical Research, National Institutes of Health, Ministry of Health, Malaysia |
| International Pandemic Preparedness Secretariat (IPPS) |
| International Readiness for Preventing Infectious Viral Disease (INTREPID Alliance) |

| IQVIA |
| --- |
| Johnson & Johnson |
| Liverpool School of Tropical Medicine |
| Medicines and Healthcare products Regulatory Agency (MHRA) |
| Medicines Patent Pool (MPP) |
| Midwest Antiviral Drug Discovery (AViDD) Center |
| National Academy of Medicine |
| National Center for Advancing Translational Sciences/ US National Institute of Health (NCATS/NIH) |
| Novartis |
| Office of Pandemic Preparedness and Response Policy (OPPR), White House |
| Pandemic Institute, University of Liverpool |
| Pandemic Preparedness Platform for Health and Emerging Infectious Response (PANTHER) |
| Peter Doherty Institute of Infection and Immunity |
| Public Health Agency of Canada |
| Rapidly Emerging Antiviral Drug Development Initiative (READDI) |
| Roche |
| Science for Africa Foundation (SFA) |
| Shionogi Inc |
| SPRIND (German Federal Agency) |
| Stanford University/ ViRx@Stanford |
| The HEVER Group |
| Universidade Federal de Minas Gerais (UFMG) |
| UK Government Office of Science |
| Unitaid |
| Univercells |
| University College London |
| University of British Columbia |
| University of Leuven |
| University of Liverpool |
| University of Melbourne |
| University of North Carolina Chapel Hill |
| University of Oxford |
| University of Toronto & University Health Network |
| Unizima |
| Wellcome |
| World Health Organization |
